# Healthcare managers’ perspectives on direct health facility financing in Tanzania

**DOI:** 10.1371/journal.pgph.0003772

**Published:** 2025-05-28

**Authors:** Kassimu Tani, Sally Mtenga, Günther Fink, Fabrizio Tediosi

**Affiliations:** 1 Ifakara Health Institute, Dar es Salaam, Tanzania; 2 Swiss Tropical and Public Health Institute, Basel, Switzerland; 3 University of Basel, Basel, Switzerland; The AgaKhan University, PAKISTAN

## Abstract

Health systems in low- and middle-income countries often face severe resource constraints and are implementing reforms to improve accountability and efficiency. Healthcare managers and governance structures are key for the successful implementation of these reforms. This study aimed to examine the implementation of the Direct Health Facility Financing (DHFF), focusing on the perspectives of health facility in-charges and members of Council Health Management Teams (CHMTs). This study employed a cross-sectional web-based questionnaire administered to all heads of public health facilities and members of CHMTs in the Kilimanjaro and Morogoro. First, we analyzed the demographics of healthcare managers, characteristics of health facilities, and the reported implementation of DHFF governance. We then performed multivariate ordered logistic regressions analyses to examine the associations between healthcare managers’ perceptions of DHFF implementation, reported changes, and resource allocation changes while controlling for health managers and facility characteristics. 348 health managers participated in the study. 23% of health facility in-charges had received DHFF-related training in the previous 12 months. 76% reported that supportive supervision explicitly includes DHFF considerations. 92% of CHMTs reported a decrease in administrative workload following DHFF implementation, compared to 80% of facility managers. Positive perceptions of autonomy in planning, budgeting and fund management were widespread (88% facility in-charge and 95% CHMTs). Health managers with higher levels of education reported positive perceptions of strong DHFF governance. Urban facilities were more likely to report higher overall DHFF governance. As the number of staff trained in DHFF increased, the positive perceptions of challenges also increased. Facility managers with a university degree perceived a successful impact of DHFF in increasing their financial resources. This study suggests that the implementation of the DHFF was positively received by health facility managers. The DHFF appears to have led to improvements in resource mobilization and financial incentives potentially contributing to overall efficiencies.

## Introduction

Health systems in low- and middle-income countries are implementing reforms aiming to improve efficiency [1–4]. The implementation of reforms requires sound governance and management of health systems. In particular, granting greater financial autonomy to healthcare institutions has been proposed in several countries as a strategy to improve efficiency [1,5,6].

Effective governance mechanisms provide the necessary framework for strategic decision-making, resource allocation and accountability in the health sector [3,7,8]. In addition, skilled management capacity is essential for translating reformed agendas into tangible actions on the ground, ensuring that policies are effectively implemented and outcomes are realized [9–11]. One promising avenue for reform is to give health facilities greater financial autonomy. By granting these institutions greater control over their financial resources, they can tailor their spending to the specific needs of their patient populations and operational contexts [12]. This autonomy could foster innovation, responsiveness, and accountability at the local level, leading to improvements in service delivery and overall system efficiency.

Many countries are actively considering or implementing reforms to decentralize financial decision-making within their health systems [3–5,13,14]. This includes mechanisms such as shifting from centralized budget allocations to facility-based financing models, which empower healthcare providers to manage their budgets autonomously [1,15–17]. While the decentralization of financial authority holds great promise, it also requires careful planning and monitoring to mitigate potential risks. Ensuring equitable distribution of resources, safeguarding against corruption or misuse of funds, and maintaining oversight mechanisms to uphold quality standards are essential considerations in this process [16,18,19].

Like many other low- and middle-income countries, Tanzania has a decentralized health system [5,20]. Its health services are managed at national, regional, and district levels. Local government authorities (LGAs) have significant control over health services within their jurisdictions [21,22]. The health system is financed through general taxation, health insurance schemes, out of pocket payments and donors’ contributions [23,24]. Direct Health Facility Financing (DHFF) was introduced in 2017/18 financial year and funds are transferred directly to the relevant health facilities without intermediaries, while higher levels as ministry of health, regions and districts only receive notifications from the Ministry of Finance confirming the amounts sent to the health facility bank account [16,25,26]. Before reforms, funding for health facilities were channeled through districts/councils [27].

### Direct health facility financing

Tanzania has historically faced significant challenges in health financing, including limited transparency in the allocation and utilization of resources at health facilities, coupled with inadequate accountability for funds generated at the facility level [28]. In response to these issues, the DHFF initiative was introduced during the Joint Health Sector Review in December 2016. This reform redefined health facilities as autonomous entities with the authority to independently manage their finances [27]. The DHFF initiative finances a wide range of health-related needs, including the procurement of health commodities, support for locally employed staff, facility renovations, and operational costs. It channels funds directly into facility bank accounts from various sources, including the Health Basket Fund, insurance reimbursements, fee-for-service payments, locally collected revenues, central government grants, donors, NGOs, and other contributors [16,29,30]. The primary goal of the DHFF was to enhance health system performance by creating a direct link between financing and service delivery. The initiative sought to foster greater autonomy, transparency, and accountability at the facility level by mandating that all transactions be recorded and reported through the Facility Financing and Accounting Reporting System (FFARS) [4,15–17,26,27,29]. By entrusting facility management with the stewardship of these resources, the DHFF seeks to cultivate a culture of accountability and ownership, thereby promoting more prudent and responsible use of financial resources [17,26,30]. This comprehensive approach should facilitate the efficient allocation of resources across various operational areas, including staffing, procurement of medical supplies and equipment, maintenance of infrastructure, and other essential components critical to effective service delivery. By empowering health facilities to strategically manage their finances, the DHFF aims to optimize resource utilization, improve service quality, and ultimately improve health outcomes for the population.

Several studies have highlighted the central role of DHFF in improving the availability of health commodities and services [15,16,22], albeit subject to the provision of adequate training on implementation protocols and the facilitation of supportive oversight mechanisms [31]. However, it is imperative to recognize that successful implementation of DHFF may be subject to variations in the capacity of health managers, potentially resulting in different standards of health care delivery that could affect the availability and quality of services [15–17,26,27,29].

The primary objective of this study was to examine the implementation of the DHFF in Tanzania, focusing on the perspectives of key management actors, such as health facility in-charges and members of council health management teams (CHMTs). The involvement of these actors is crucial, as it allows for tailored services that better meet local needs, thereby improving both quality and uptake [32]. However, weak health facility management can hinder effective implementation, resulting in poor health system functioning and reduced patient access to care. By examining the DHFF implementation from the perspective of key management actors, this study aimed to provide insights into governance factors and their impact on implementation.

## Methods

### Study setting

This study was conducted in two regions of Kilimanjaro and Morogoro. The Kilimanjaro region comprises seven districts, namely Hai, Moshi, Moshi rural, Mwanga, Rombo, Same, and Siha. Whereas Morogoro region encompasses nine districts: Morogoro rural, Morogoro urban, Mvomero, Gairo, Ifakara town, Malinyi, Ulanga, Kilombero, and Kilosa. The survey was conducted within Tanzania’s primary health care (PHC) system which operates under two separate ministries. The Ministry of Health (MoH), which is responsible for resources mobilization, policy formulation and the management of regional, zonal, and national hospitals, whereas the President’s Office – Regional Administration and Local Government (PO-RALG), oversees the implementation of PHC services. At the time of the study Kilimanjaro had 248 PHC facilities and Morogoro had 313.

### Study population

This study focuses on health facility managers and the members of CHMTs. The health facility in-charges responsibilities include overseeing the flow of resources from various sources such as out-of-pocket payments, health insurance schemes, and government. The CHMT members are responsible for coaching, mentoring, and ensuring that health facilities are performing well. Together, they constitute the study population.

### Study design

This study used a cross-sectional, quantitative survey design, with a web-based questionnaire sent via WhatsApp to collect data from all public health facility in-charges and CHMTs in each district of the Kilimanjaro and Morogoro regions.

We developed the questionnaire using an iterative approach. We first reviewed the relevant literature on DHFF [1,17,27,33]. We then sought input from senior health system researchers with experience of health services financing in Tanzania. We incorporated their recommendations to refine the questionnaire. The revised version was then distributed to a selected group of healthcare managers (six) in Morogoro municipal council via their WhatsApp numbers to pilot the questionnaire. We considered and responded to the feedback we received, making the necessary adjustments to ensure the validity of the questionnaire.

### Sampling strategy and sample size

We purposively selected all the districts of the Kilimanjaro and Morogoro regions because this study was embedded within the larger study [34,35].

At districts/councils and health facilities levels we employed a comprehensive census-sampling approach, targeting all active public healthcare managers within health facilities and district/council administrations [36–38]. The total number of healthcare managers, was estimated based on the registered number of public health facilities (dispensaries, health centres, and hospitals) within the selected districts/councils, alongside the projected number of CHMTs members. As per the established protocol, CHMTs consists of 24 core and co-opted members. This information was sourced from the Ministry of Health’s staffing level document for health service facilities, health training institutions and agencies (2014–2019 revision) [39].

Recruitment was conducted through existing local WhatsApp groups of health managers. Assuming a hypothetical 100% response rate, the maximum number of potential respondents was 849. However, prior research on email and web-based surveys suggests response rates typically range from 5% to 50% [40–43] (Table A in S1 Appendix).

### Data collection

The online survey was distributed between 3^rd^ July, 2023 and 2^nd^ September, 2023–561 targeted health facility ‘in-charges’ at the health facility level and 288 members of the CHMTs at the district level (Table A in S1 Appendix). After receiving approval and introduction letters from president office, regional administration and local government (PORALG) to the regional and districts levels, we contacted the district medical officers (DMOs) and then district health secretaries (DHS) via phone call and WhatsApp to brief them about the research and the survey in general. The DHS helped distribute the survey links to the WhatsApp groups in their administrative areas, and provided the follow-up reminders to encourage completion. We used the Open Data Kit (ODK) and Enketo to collect the data from all health managers who participated in the study. We reimbursed the respondents for the airtime used to complete the questionnaire with an amount equivalent to 4 USD (10,000/ = TZS) sent directly to their mobile money account. However, they did not receive any other form of compensation. Importantly the reimbursement did not influence their responses, as the funds were transferred only after they had completed and submitted the survey.

### Questionnaire and variable descriptions

The survey included background information on the health facilities and districts involved, as well as detailed profiles of the respondents (Table C in S1 Appendix). Participants were queried about governance challenges and successes associated with DHFF, and its role in financing and thus sustaining their health care facilities. The survey included the following sections:

1. Demographic information about the respondents; age, gender, experience in years in a management position, and cadre.
2. Information on the health facility where the respondent worked most of the time, including health facility location (rural/urban), level and information on the implementation of DHFF.
3. Questions about awareness of policies or guidelines to implement DHFF. We developed a list of awareness questions that may have been implemented in health facilities, regarding DHFF.
4. Questions about challenges faced by health managers since the start of the DHFF implementation. We inquired whether they had experienced administrative burdens, experienced problem in administration of funds, autonomy in planning, budgeting and managing funds, if the received funds cater all planned activities, if they were able to purchase inputs on time, if there were delays in decision-making regarding inputs purchases, and whether the facility had all necessary inputs and sufficient space to provide services.
5. Questions on health managers’ perceptions of the implementation of DHFF scheme.

To generate an overall perception on governance implementation, we calculated two separate sub-scores: one for the perceived challenges of DHFF implementation, and another for perceived changes in available resources. This was done by summing the responses to each question, resulted in score categories ranged from 0 to 3 for challenges and 0–7 for changes in resources.

We used 18 perception-based questions to assess the implementation of DHFF. These questions were rated on a five-point Likert scale, ranging from 1, indicating ‘strongly disagree’, to 5, signifying ‘strongly agree’. To maintain uniformity and consistency, we applied reverse coding to negatively worded questions, so that a rating of 5 consistently represented a positive attitude.

The key dependent variables included overall perceptions of DHFF implementation, perceived implementation challenges, resource change scores, and Likert scale responses of each question from health managers. To ensure methodological rigor, we controlled for a range of independent variables: age categories of health managers, gender, cadre, educational level, years of experience in a management position, locality of the facility, health facility level, number of health workers in the facility, and number of staff trained in DHFF.

### Analysis

The analysis involved both descriptive and analytical methods. Initially, we tabulated information on the demographics of healthcare managers and their primary responsibilities to identify any variations or patterns. Subsequently we examined health managers’ perspectives on the DHFF program, capturing nuanced insights into various health system dimensions such as coaching and mentoring practices, rules and regulatory adherence, institutional autonomy, and the availability of services. To further investigate the factors influencing DHFF implementation success, we conducted multivariate ordered logistic regressions. Our analysis aimed to explore the relationship between healthcare managers’ perceptions of DHFF implementation, perceived challenges, and changes in resource allocation, while controlling for specific characteristics of health managers and their facilities. Given that the dependent variables consisted of ordered score values, and exceeded two categories, we employed ordered logistic regression. We constructed two models for each outcome indicator: the first utilized ordered logistic regression to assess implementation scores, aiming to identify predictors influencing health managers’ perceptions of DHFF implementation. The second model employed ordered logistic regression to analyze health managers’ Likert scale responses, identifying predictors influencing perceptions of the DHFF program. This model was chosen as it accounted for the ordinal nature of the dependent variable and it assumes that the relationship between each pair of outcome groups is statistically similar. The proportional odds assumption was tested and confirmed using the Brant test (Table D in S1 Appendix).

To evaluate the adequacy of our model specification, we employed the “linktest” command in Stata. This test assesses the functional form and specification of the model by examining the relationship between predicted probabilities and the observed outcomes. By doing so it ensures that the model accurately captures the relationship between the independent variables and the outcome variable.

Recognizing that our respondents were nested within districts having a distinct District Medical Officer (DMO), we employed clustering in our analysis. Clustering adjusts for potential within-group correlation, resulting in more accurate standard errors and inferential statistics. All statistical analyses were conducted using Stata18, and statistical inference was based on a significance level of 5% (α = 0.05).

### Ethics approval and consent to participate

Ethical approval for this study was obtained from the Ifakara Health Institute (IHI) Institutional Review Board (IHI/IRB/AMM/No:19–2022) and the Tanzanian National Institute for Medical Research (NIMR/HQ/R.8b/Vol.1/1063). All activities had been performed in accordance with IHI and NIMR guidelines and regulations on human research. The ministry of local government was informed and provided permission for conducting surveys within their respective districts. The district level authorities provided permission to contact district health secretaries who assisted in the distribution of the surveys to the health managers at their local district. Written informed consent was obtained prior to the start of the survey, and respondents were required to confirm their understanding and intent to proceed by ticking three boxes, providing written confirmation, and signing via the smartphone used to complete the survey in order to proceed to the survey. The survey was end-to-end encrypted to ensure respondents’ privacy, and data was sent to a secure server at the Ifakara Health Institute.

### Inclusivity in global research

Additional information regarding the ethical, cultural, and scientific considerations specific to inclusivity in global research is included in the Supporting Information (S1 Checklist).

### Role of the funding source

The funders had no role in study design, data collection and analysis, decision to publish, or preparation of the manuscript.

## Results

### Demographic characteristics of study participants

A total of 348 health managers participated in the study, comprising 144 members of the CHMTs and 204 health facility in-charges. The majority of CHMT respondents were female (52%), while health facility in-charges were predominantly male (62%). Of the CHMT participants, 21% were medical doctors, while health facility in-charges were predominantly clinical officers (58%) (Table 1). Most CHMT members (61%) held a university degree, while health facility in-charges (66%) primarily held diplomas. More than 50% of health managers in both CHMT and health facility in-charge roles reported having over 5 years of experience in their respective positions (Table 1). Table B in S1 Appendix shows additional information on the services provided and the staffing levels of the facilities where respondents work.

**Table 1:** Respondent characteristics by role.

Variables	CHMT (n = 144)			Facility in-charge (n = 204)		
	Total	Kilimanjaro	Morogoro	Total	Kilimanjaro	Morogoro
	n (%)	n (%)	n (%)	n (%)	n (%)	n (%)
**Gender**						
Female	75 (52.1)	36 (60.0)	39 (46.4)	78 (38.2)	38 (38.4)	40 (38.1)
Male	69 (47.9)	24 (40.0)	45 (53.6)	126 (61.8)	61 (61.6)	65 (61.9)
**Age category**						
Below 35	53 (36.8)	21 (35.0)	32 (38.1)	146 (71.6)	79 (79.8)	67 (63.8)
36 - 45	65 (45.1)	24 (40.0)	41 (48.8)	40 (19.6)	11 (11.1)	29 (27.6)
above 45	26 (18.1)	15 (25.0)	11 (13.1)	18 (8.8)	9 (9.1)	9 (8.6)
**Cadre**						
Enrolled Nurse	1 (0.7)	0 (0)	1 (1.2)	11 (5.4)	5 (5.1)	6 (5.7)
Registered Nurse	11 (7.6)	4 (6.7)	7 (8.3)	18 (8.8)	13 (13.1)	5 (4.8)
Nurse Officer	17 (11.8)	8 (13.3)	9 (10.7)	6 (2.9)	4 (4.0)	2 (1.9)
Assistant Clinical Officer	0 (0.0)	0 (0.0)	0 (0.0)	20 (9.3)	9 (9.1)	11 (10.5)
Clinical Officer	5 (3.47)	2 (3.3)	3 (3.6)	117 (57.8)	54 (54.6)	63 (60.0)
Assistant Medical Officer	6 (4.17)	4 (6.7)	2 (2.4)	6 (2.9)	2 (2.0)	4 (3.8)
Medical officer	30 (20.83)	11 (18.3)	19 (22.6)	24 (11.8)	10 (10.1)	14 (13.3)
Laboratory technician	8 (5.56)	4 (6.7)	4 (4.8)	2 (1.0)	2 (2.0)	–
Medical specialist	3 (2.08)	2 (3.3)	1 (1.2)	–	–	–
Pharmacist	6 (4.17)	2 (3.3)	4 (4.8)	–	–	–
Health secretary	15 (10.42)	5 (8.3)	10 (11.9)	–	–	–
Social officer	9 (6.25)	2 (3.3)	7 (8.3)	–	–	–
Environment officer	17 (11.81)	8 (13.3)	9 (10.7)	–	–	–
Nutritionist	7 (4.86)	3 (5.0)	4 (4.8)	–	–	–
DHFFco	3 (2.08)	2 (3.3)	1 (1.2)	–	–	–
Technician	6 (4.17)	3 (5.0)	3 (3.6)	–	–	–
**Professional**						
Clinical	44 (30.6)	19 (31.7)	25 (29.8)	167 (81.9)	75 (75.7)	92 (87.6)
Non clinical	100(69.4)	41 (68.3)	59 (70.2)	37 (18.1)	24 (24.3)	13 (12.4)
**Education level**						
Certificate	1 (0.7)	0 (0)	1 (1.2)	37 (18.1)	20 (20.2)	17 (16.2)
Diploma	22 (22.2)	15 (25.0)	17 (20.2)	135 (66.2)	66 (66.7)	69 (65.7)
Advanced diploma	10 (6.9)	4 (6.7)	6 (7.1)	7 (3.4)	4 (4.0)	3 (2.9)
University degree	88 (61.1)	34 (56.7)	54 (64.3)	25 (12.3)	9 (9.1)	16 (15.2)
MPH/ MSc	9 (6.3)	7 (11.7)	6 (7.1)	–	–	–
**Management years of Experience**						
Less than 5 years	66 (45.8)	29 (48.3)	37 (44.1)	99 (48.5)	59 (59.6)	40 (38.1)
Above 5 years	78 (54.2)	31 (51.7)	47 (55.9)	105 (51.5)	40 (40.4)	65 (61.9)
**Health facility level**						
Dispensary	–	–	–	155 (76.0)	74 (74.8)	81 (77.1)
Health Centre	–	–	–	46 (22.5)	23 (23.2)	23 (21.9)
Hospital	–	–	–	3 (1.5)	2 (2.0)	1 (1.0)
**Trained on DHFF implementation**						
Yes	44 (30.6)	40 (66.7)	60 (71.4)	47 (23.0)	24 (24.2)	82 (78.1)
No	100 (69.4)	20 (33.3)	24 (28.6)	157 (77.0)	75 (75.8)	23 (21.9)

### DHFF implementation practices

Examining DHFF implementation practices reveals that 23% of health facility in-charges received DHFF-specific training sessions in the last 12 months, while 79% reported that their accounting staff had received relevant training. Moreover, 76% indicated that supportive supervision explicitly includes DHFF considerations (Table 2). Notably, most facility in-charges reported minimal technical problems involving the receipt, accounting, and administration of funds, highlighting a relatively smooth financial management process.

**Table 2:** Frequencies and percentage of reported implementation of DHFF (Facility in-charge).

DHFF implementation practices	Facilities n (%)
DHFF training/mentorship sessions in last 12 months	47 (23.0)
Accounting managing staff oriented on DHFF implementation	161 (78.9)
During supportive supervision from CHMT did you discuss DHFF	155 (75.9)
Finance and accounting staff are adequately qualified and experienced with DHFF	143 (70.1)
Experienced problems involving the receipt, accounting and/or administration of funds	57 (27.9)
**DHFF implementation policy requirement**	
Guidelines and operational manuals for DHFF and FFARS	161 (78.9)
Have Annual Health Facility Plan Guideline	196 (96.1)
Have annual health facility plan - HFP	196 (96.1)
Convene a management meeting	181 (88.7)
Involved other staff in implementation and decision making on DHFF	192 (94.1)
**DHFF implementation outcome**	
Received funds as requested in Budget	90 (44.1)
Funds meets operational requirements as stipulated in the budget	56 (27.5)
Receive timely the funds from iCHF, NHIF and Basket fund	83 (40.7)

A significant proportion of facility in-charges reported the availability of operational guidelines and manuals (78%), health facility plans (96%), and the regular convening of management meetings (88%). Collaborative decision-making processes were prevalent, with 94% of decisions involving colleagues (Table 2). Less than half of the facility in-charges reported receiving funds as stipulated in the budget (44%), with 27% indicating that the funds met operational requirements. Additionally, only 40% reported timely receipt of funds, suggesting a potential area for improvement in the implementation timeline (Table 2).

The healthcare managers who responded to the survey revealed a notable reduction in administrative burden when funds were received directly at health facilities. 92% of CHMT members reported a decline in administrative burden, compared to approximately 80% among facility in-charges. Additionally, more than 95% of facility in-charges reported actively using government procurement systems for ordering medical products and reporting expenditures, showcasing a widespread adoption of standardized processes (Table 3).

**Table 3:** Frequencies and percentages of reported component relating to rules, regulation, and autonomy.

Variables	CHMT n (%)	In-charge n (%)
Flow of funds direct to the health facilities decrease the funds administrative burden	133 (92.4)	163 (79.9)
Use government procurement systems to purchase all supplies needed to service the health beneficiaries	140 (97.2)	194 (95.1)
The facilities managements have direct control of all finances resources	128 (88.9)	169 (82.8)
The facilities has full autonomy on the planning of all the activities	132 (91.7)	181 (88.7)
The facilities has full autonomy on budgeting	134 (93.1)	184 (90.2)
The facilities has full autonomy on managing all funds received or raised at facility	137 (95.1)	191 (93.6)
The facilities experienced problems in the past involving the receipt, accounting and/or administration of funds	67 (46.5)	57 (27.9)
The facilities receive fund to cater all the planned activities	33 (22.9)	90 (44.1)
The facilities purchased all items required for services provision on time	68 (47.2)	101 (49.5)
The facilities you manage have all the medicine and supplies required to provide services	84 (58.3)	77 (37.8)
The reforms made to disbursement of fund direct to the facility has reduced delays in receiving funds to pay for daily operation’s needs	109 (75.7)	104 (50.9)
Acted to ensure staff in health facility are accountable on services provision	129 (92.1)	–
The health facility in-charges sought guidance from you on DHFF operations in the last 6 month	97 (67.8)	–
Worrying about the ability of health facility in-charges capacity to manage DHFF	43 (29.7)	–

Regarding direct control of financial resources, a majority of CHMT members (88.9%) and facility in-charges (82.8%) agreed on the importance of this aspect. In terms of autonomy in planning, budgeting, and fund management, a positive perception was prevalent among health managers, ranging from 88% to 95% (Table 3).

When assessing resources at health facilities, a notable percentage of CHMT members (22.9%) and facility in-charges (44.1%) acknowledged insufficiencies in catering to all needs. Slightly less than half of all respondents reported the ability to purchase required inputs on time, while more than half of CHMT members perceived health facilities to have adequate resources for purchasing all required medicines, and only 37% of facility in-charges shared this view (Table 3).

About half of facility in-charges perceived that the implementation of DHFF had not reduced delays in receiving funds for daily operations. However, this perception contrasted with that of CHMT members, of whom three-quarters perceived that DHFF had reduced delays. Notably, almost all CHMT members (92%) reported taking actions to ensure facility in-charges were accountable for resources they received. However, more than 30% noted that facility in-charges did not seek guidance when needed. The majority of CHMT members expressed confidence in the capability of health facility in-charges to perform their roles effectively (Table 3).

**Fig 1:**
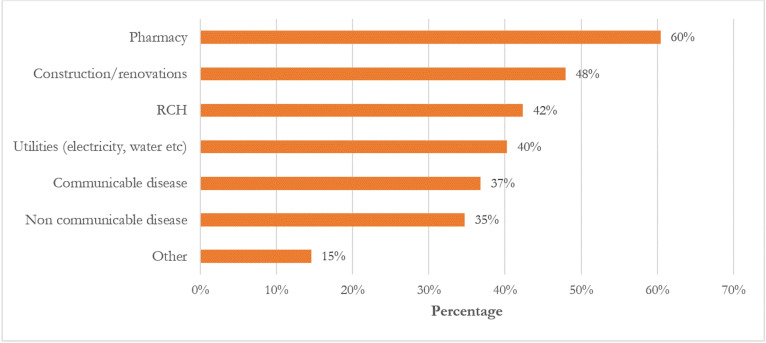
Directive regarding governance of DHFF on activities.

More than 60% of facility in-charges reported that funds allocated for the purchase of medicines are subject to binding guidelines. Conversely, fewer respondents reported similar mandatory guidelines for other expenditure categories such as construction (47.0%), reproductive and child health (RCH) initiatives (42%), and communicable and non-communicable disease management (less than 40%) (Fig 1).

### Ordered logistic regression of health facility in-charges views on DHFF governance, challenges, and changes on available resources

In the multivariate analysis (Table 4), we accounted for various factors including gender, age category, education level, cadre, experience as a healthcare manager, health facility locality, and health facility size measured by the number of staff and beds, the results revealed significant associations with DHFF implementation scores.

**Table 4:** Multivariate Ordered Logistic Regression models of Predictors of Reported DHFF governance, perceived challenges and resources changes by Facility in-charges.

Variable	All DHFF governance implementation scores		DHFF perceived challenges score		DHFF resources changes score	
	Adjusted Odd ratio (CI)	P-value	Adjusted Odd ratio (CI)	P-value	Adjusted Odd ratio (CI)	P-value
Gender						
Male	–	–	–	–	–	–
Female	1.13 (0.56-2.26)	0.72	1.10 (0.61-1.97)	0.74	1.25 (0.65-2.40)	0.50
Age category						
Below 36	–	–	–	–	–	–
36-45	0.58 (0.24-1.38)	0.22	0.66 (0.35-1.24)	0.19	0.65 (0.31-1.37)	0.26
Above 45	0.41 (0.17-0.92)	0.03**	0.22 (0.05-0.86)	0.03**	0.47 (0.16-1.38)	0.17
Education						
Certificate	–	–	–	–	–	–
Diploma	1.56 (0.76-3.22)	0.22	0.87 (0.43-1.75)	0.71	1.75 (0.84-3.64)	0.13
Advance diploma	6.15 (1.32-28.61)	0.00***	0.24 (0.01-16.78)	0.51	34.76 (2.48-486.88)	0.01**
Degree	3.25 (0.69-15.18)	0.13	0.08 (0.01-13.70)	0.34	8.25 (0.36-184.65)	0.18
Cadre						
Laboratory technicians	–	–	–	–	–	–
Nurses	8.58 (2.67-27.54)	0.00***	6.72 (1.21-37.31)	0.02**	8.25 (1.70-40.04)	0.01**
Clinical officer	4.09 (1.04-16.01)	0.04**	9.12 (1.24-66.87)	0.03**	3.31 (0.48-22.84)	0.22
Medical officer	1.21 (0.44-3.31)	0.71	39.41 (2.31-671.57)	0.01**	0.54 (0.11-2.64)	0.45
Experience (years)	1.02 (0.97-1.07)	0.38	1.01 (0.91-1.10)	0.92	1.02 (0.96-1.10)	0.42
Training sessions	1.25 (0.93-1.68)	0.12	0.89 (0.60-1.31)	9.55	1.14 (0.37-1.49)	0.31
Locality						
Rural	–	–	–	–	–	–
Urban	3.19 (1.73-5.88)	0.00***	2.73 (1.29-5.79)	0.01**	0.64 (1.46-4.78)	0.01**
Number of observation bed	1.02 (0.99-1.05)	0.08	1.04 (1.01-1.08)	0.01**	1.02 (0.98-1.05)	0.21
Number of staffs	0.97 (0.95-0.98)	0.01**	0.98 (0.94-1.03)	0.60	0.96 (0.94-0.98)	0.01**
Staffs trained on DHFF	1.16 (0.83-1.61)	0.38	1.62 (1.04-2.51)	0.02**	1.08 (0.76-1.54)	0.64

* p < 0.05** p < 0.01*** p < 0.001.

For health facility in-charges aged above 45 years, they reported a 59% lower overall DHFF implementation score (OR= 0.41, CI = 0.17-0.92). The decline was even more pronounced for perceived challenges scores, which were 78% lower (OR=0.22, CI = 0.05-0.86).

Comparatively, health managers with higher education levels than the reference group reported a more positive opinion on DHFF governance. Specifically, those with a diploma (OR=1.56, CI = 0.76–3.22), advanced diploma (OR=6.15, CI = 1.32–28.61), and/or a university degree (OR=3.25, CI = 0.69 –15.18) were more likely to report strong governance.

Distinct cadres exhibited varied responses. Using laboratory technicians as a reference group; nurses (OR=8.58, CI = 2.67-27.54) and clinical officers (OR=4.09, CI = 1.04 -16.01) were more likely to report positive DHFF governance (Table 4).

Health facility in-charges in urban areas were more likely to report higher overall DHFF governance (OR=3.19, CI = 1.73-5.88). In disaggregated models, the urban health facility in-charges were more likely to report higher scores for perceived DHFF challenges (OR=2.73, CI = 1.29-5.79) but were 36% less likely to agree that resources improved (OR=0.64, CI = 1.46 - 4.78) (Table 4).

Considering facility size in relation to the number of staff, a larger staff complement was found to be associated with a 3% decrease in the likelihood of health facility in-charges reporting good DHFF implementation (OR=0.97, CI = 0.95-0.98). On the contrary, an increased number of staff trained in DHFF correlated with higher scores in DHFF governance, positive perceived challenges scores, and changes in resources. Perceived challenges scores were statistically significant in this regard (OR=1.62, CI = 1.04-2.51) (Table 4).

### Ordered logistic regression of health facility in-charges and CHMT member views on DHFF impacts

Tables 5 and 6 present the results of the multivariate analyses examining the perceptions of health facility in-charges and CHMT members regarding the impact of DHFF on various aspects of service provision, including efficiency, availability of health services, resource mobilization, and financing. Overall, the results reveal mixed responses.

**Table 5:** Multivariate Ordered Logistic Regression models of Predictors of Perceived DHFF implementation Impact by Facility in-charges.

	The introduction of DHFF and its implementation to the moment in my place of work has improved efficiency in provision of services	The introduction and implementation of DHFF creates an environment in which health facilities are more likely to respond to financial incentives	DHFF has improved the availability of health services	DHFF has improved mobilization of resources	DHFF has facilitated financing of health services at health facilities
**Variable**	**AOR(CI)**	**AOR(CI)**	**AOR(CI)**	**AOR(CI)**	**AOR(CI)**
Gender					
Male	–	–	–	–	–
Female	0.94 (0.47-1.87)	0.86 (0.50-1.47)	0.85 (0.41-1.78)	0.82 (0.50-1.35)	0.63 (0.32-1.21)
Age	1.05 (0.97-1.15)	1.08 (0.97-1.19)	1.05 (0.97-1.13)	1.11 (1.02-1.19)**	1.06 (0.99-1.14)*
Education					
Certificate	–	–	–	–	–
Diploma	1.17 (0.58-2.36)	1.12 (0.59-2.14)	1.47 (0.63-3.38)	0.96 (0.40-2.32)	1.49 (0.70-3.19)
Advance diploma	0.79 (0.05-10.61)	0.37 (0.03-3.89)	2.27 (0.69-74.58)	3.13(0.08-117.80)	3.06 (0.24-38.06)
Degree	4.51 (0.54-37.4)	2.00 (0.16-25.11)	8.84 (0.44-174.08)	10.26 (0.41-255.32)	17.78 (2.33-135.22)**
Cadre					
Laboratory technicians	–	–	–	–	–
Nurses	1.19 (2.51-5.67)***	4.74 (8.90-2.52)***	0.19 (0.03-1.19)	0.17 (0.02-1.74)	0.08 (0.01-0.72)**
Clinical officer	2.17 (5.58-8.45)***	6.72 (1.44-3.13)***	0.23 (0.04-1.42)	0.32 (0.02-4.04)	0.09 (0.01-0.91)**
Medical officer	2.99 (3.30-2.72)***	1.17 (1.02-1.34)***	0.16 (0.02-0.91)**	0.07 (0.01-0.38)**	0.06 (0.01-0.31)**
Experience (years)	0.97 (0.89-1.05)	0.95 (0.86-1.05)	0.96 (0.89-1.05)	0.90 (0.84-0.96)**	0.96 (0.89-1.02)
Training sessions	1.41 (0.89-2.24)	1.28(0.86-1.89)	1.48 (0.84-2.62)	1.11 (0.63-1.94)	0.90 (0.52-1.55)
Facility level					
Dispensary	–	–	–	–	–
Health centre	0.24 (0.08-0.77)**	0.38 (0.11-1.42)	0. 20 (0.07-0.54)**	0.24 (0.05-1.12)	0.14 (0.02-1.10)
Hospital	0.16 (0.01-3.28)	0.21 (0.01-5.02)	0.07 (0.01-1.03)*	0.11 (0.01-2.81)	0.37 (0.01-10.16)
Locality					
Rural	–	–	–	–	–
Urban	1.41 (0.594-3.32)	1.11 (0.50-2.43)	1.10 (0.64-1.87)	1.30 (0.54-3.12)	1.17 (0.56-2.43)
Number bed	1.03 (1.00-1.01)**	1.03 (1.00-1.06)**	1.06 (1.03-1.09)***	1.05 (1.01-1.09)**	1.05 (1.01-1.09)**
Number of staffs	0.98 (0.94-1.02)	0.98 (0.93-1.04)	0.98 (0.94-1.03)	0.98 (0.93-1.03)	0.96 (0.91-1.02)
Staffs trained on DHFF	1.18 (0.79-1.73)	1.11 (0.72-1.67)	1.08 (0.78-1.51)	1.13 (075-1.68)	1.40 (0.93-2.09)

* p < 0.05** p < 0.01*** p < 0.001.

**Table 6:** Multivariate Ordered Logistic Regression models of Predictors of Reported Impact of DHFF implementation by CHMTs.

	The introduction of DHFF and its implementation and governance to the moment in my place of work has improved efficiency in provision of services	The introduction and implementation of DHFF creates an environment in which health facilities are more likely to respond to financial incentives	DHFF has improved the availability of health services	DHFF has improved mobilization of resources	DHFF has facilitated smooth financing of health services at health facilities
**Variable**	**AOR(CI)**	**AOR(CI)**	**AOR(CI)**	**AOR(CI)**	**AOR(CI)**
Gender					
Male	–	–	–	–	–
Female	0.91 (0.40-2.08)	0.69 (0.36-1.33)	1.06 (0.47-2.36)	1.06 (0.47-2.34)	0.71 (0.26-1.94)
Age	0.99 (0.94-1.04)	1.02 (0.98-1.06)	1.01 (0.95-1.05)	0.98 (0.94-1.02)	1.01 (0.94-1.07)
Education					
Certificate	–	–	–	–	–
Diploma	1.07 (0.32-3.58)	1.29 (0.71-2.34)	1.49 (0.60-3.68)	1,35 (0.84-2.16)	2.11 (0.89-4.99)*
Advance diploma	2.55 (0.34-18.61)	1.05 (0.30-3.63)	4.76 (0.77-26.18)*	4.49 (1.02-19.76)**	5.79 (0.77-43.64)
Degree	1.24 (0.60-2.55)	1.41 (0.76-2.62)	1.63 (0.61-4.33)	1.88 (0.96-3.67)*	1.56 (0.48-5,05)
Master Degree	0.46 (0.09-2.22)	1.02 (0.17-6.05)	0.61 (0.06-6.52)	1.25 (0.15-9.91)	1.51 (0.22-10.06)
Cadre					
Non-Medical	–	–	–	–	–
Nurses	1.01 (0.57-1.77)	0.61 (0.39-0.96)**	0.89 (0.44-1.79)	1.28 (0.71-2.31)	1.11 (0.42-2.89)
Physician	0.61 (0.27-1.33)	0.57 (0.22-1.47)	0.57 (0.27-1.22)	0.83 (0.39-1.78)	1.30 (0.58-2.88)
Experience (years)	0.98 (0.93-1.03)	0.97 (0.91-1.03)	1.02 (0.97-1.07)	1.03 (0.99-1.05)	1.03 (0.98-1.07)
Trained on DHFF					
No	–	–	–	–	–
Yes	1.64 (0.68-3.94)	1.33 (0.38-4.63)	1.62 (0.62-4.23)	0.96 (0.34-2.68)	1.23 (0.39 = 3.82)
Training on DHFF					
No	–	–	–	–	–
Yes	3.52 (0.93-13.31)	2.12 (0.52-8.69)	2.13 (0.51-8.78)	4.30 (1.08-17.03)**	1.68 (0.48-5.86)

* p < 0.05** p < 0.01*** p < 0.001.

There was minimal differences in views regarding whether the implementation of DHFF has improved efficiency in the provision of health services. However, facility in-charges at health centres and hospitals reported lower odds ratios for observing improved efficiency. Specifically, health centres were 24% less likely (OR=0.24, CI = 0.08-0.77), and hospitals were 16% less likely (OR=0.16, CI = 0.01-3.28) to indicate an improvement in efficiency. In contrast, taking into account health facility in-charges by cadres, there were more positive perceived changes in efficiency attributed to DHFF on service provision. In particular, nurses (OR=1.19, CI = 2.51-5.67), clinical officers (OR=2.17, CI = 5.58-8.45) and medical officers (OR=2.99, CI = 3.30-2.72) were significantly more likely to report favorable perceptions (Table 5).

Regarding the financial incentives linked to the direct funding of the health facility, managers were particularly likely to highlight positive changes. Significant differences were observed between different professional cadres, with nurses, clinical officers and medical officers more likely to report positive adjustments in financial incentives (Table 5). Conversely, CHMT members who were nurses (OR=0.61, CI = 0.39-0.96) had a less favorable view of the effectiveness of the DHFF in creating an enabling environment for health facilities to respond to financial incentives (Table 6).

In terms of improving the availability of health services, medical officer facility in-charges perceived that the DHFF had not made a significant contribution (OR=0.16, CI = 0.02 - 0.91) (Table 5). In particular, there was a significant decrease in perception of improvement in availability of health services at dispensaries and health centres by 80% (OR=0.20, CI = 0.07 - 0.54), and at hospitals by 93% (OR=0.07, CI = 0.01 - 1.03) (Table 5).

There were also discernible patterns in perceptions of the impact of the DHFF on resource mobilization. Facility managers who were older, CHMT members with advanced diploma or a university degree and those who had received DHFF training were more likely to perceive that DHFF led to increased resource mobilization. Conversely, facility in-charges with more experience in leadership positions tended to have lower perceptions of the DHFF’s success in mobilizing resources (Tables 5 and 6).

Facility in-charges with a university degree were significantly more likely to perceive the successful impact of DHFF implementation in increasing the financial resources of facility health services. Specifically, those with a university degree were 17.8 times more likely to have this perception (OR=17.8, CI = 2.33 -135.22). Taking into account the health facility in-charges by cadres, nurses (OR=0.08, CI = 0.01-0.72), clinical officers (OR=0.09, CI = 0.01-0.91) and medical officers (OR=0.06, CI = 0.01-0.31) serving as health facility in-charges had slightly lower odds ratios of perceiving the implementation of the DHFF as successful in financing health services at health facilities compared to laboratory technicians.

In addition, when considering the size of the facility, as proxied by the number of beds, there was a notable trend: the larger the health facility, the higher the odds of reporting DHFF success in several areas, including perceived improved work efficiency, facility responsiveness to financial incentives, service availability, resource mobilization and service financing (Table 5).

### Ordered logistic regression of satisfaction of health facility in-charges and CHMT members with DHFF

Tables 7–9 show the results of the multivariate analyses on the opinion of health facility in-charges and CHMT members regarding their satisfaction with DHFF implementation in terms of the formula used, DHFF design, payment flexibility, financial transparency, accountability, legal autonomy, and improvements in the management of the facility as an institution. Overall, the results revealed mixed responses.

**Table 7:** Multivariate Ordered Logistic Regression models of Predictors of Reported satisfaction of DHFF implementation by Facility in-charges.

	The formula used to decide the amount health facility receives directly from ministry of finance works optimally	I am satisfied with the way DHFF is designed	DHFF system provides a flexible approach for health facilities payment and management of the resources	DHFF has improved financial transparency	DHFF has improved financial accountability
**Variable**	**AOR(CI)**	**AOR(CI)**	**AOR(CI)**	**AOR(CI)**	**AOR(CI)**
Gender					
Male	–	–	–	–	–
Female	0.97 (0.62-1.52)	0.86 (0.36-2.04)	0.58 (0.33-1.01)*	0.69 (0.33-1.44)	0.56 (0.25-1.22)
Age	1.02 (0.98-1.06	1.04 (0.99-1.09)	1.08 (1.01-1.15)**	1.06 (1.01-1.12)**	1.05 (0.98-1.11)
Education					
Certificate	–	–	–	–	–
Diploma	1.21 (0.51-2.82)	1.05 (0.52-2.10)	1.09 (0.47-2.56)	1.27 (0.64-2.54)	1.51 (0.69-3.25)
Advance diploma	5.84 (0.57-59.13)	16.53(2.06-132-29)**	5.74 (0.29-112.96)	3.32 (1.12-9.82)**	2.47 (0.82-7.39)
Degree	3.32 (0.51-21.52)	16.12(1.88-142.89)**	8.03 (0.91-70.37)*	6.45 (1.66-25.09)**	4.53 (1.17-17.54)**
Cadre					
Laboratory technicians	–	–	–	–	–
Nurses	0.52 (0.26-1.03)*	0.17 (0.67-0.47)**	0.25 (0.46-1.40)	0.73 (0.29-1.86)	0.46 (0.18-1.21)
Clinical officer	0.51 (0.16-1.54)	0.31 (0.11-0.83)**	0.34 (0.06-1.99)	1.56 (0.54-4.47)	1.17 (0.44-3.12)
Medical officer	0.14 (0.03-0.68)**	0.04 (0.01-0.16)***	0.05 (0.01-0.21)***	0.61 (0.10-3.54)	0.58 (0.09-3.72)
Experience (years)	0.90 (0.84-0.96)**	0.89 (0.82-0.97)**	0.91 (0.84-0.98)**	0.92 (0.87-0.98)**	0.92 (0.86-0.99)
Training sessions	1.09 (0.75-1.58)	0.87 (0.57-1.32)	0.79 (0.52-1.19)	1.01 (0.57-1.77)	0.91 (0.49-1.68)
Facility level					
Dispensary	–	–	–	–	–
Health centre	0.39 (0.08-0.180)	0.30 (0.12-0.75)**	0.57 (0.188-1.73)	0.27 (0.11-0.69)**	0.24 (0.09-0.59)**
Hospital	0.69 (0.04-9.93)	2.35 (0.26-21.30)	0.48 (0.05-4.03)	3.68 (0.35-37.97)	0.34 (0.27-4.42)
Locality					
Rural	–	–	–	–	–
Urban	1.95 (0.78-4.87)	1.26 (0.71-2.23)	1.32 (0.66-2.62)	0.65 (0.29-1.47)	1.09 (0.53-2.23)
Number bed	1.01 (0.98-1.04)	1.03 (1.01-1.06)***	1.03 (1.01-1.05)**	1.02 (1.01-1.05)**	1.03 (1.01-1.06)**
Number of staffs	0.98 (0.94-1.02)	0.97 (0.93-1.01)	0.96 (0.93-0.99)**	0.96 (0.93-1.01)*	0.97 (0.95-1.01)
Staffs trained on DHFF	1.22 (0.89-1.67)	1.36 (1.05-1.77)**	1.40 (1.14-1.71)**	1.41 (1.12-1.76)**	1.41 (1.06-1.87)

* p < 0.05** p < 0.01*** p < 0.001.

**Table 8:** Multivariate Ordered Logistic Regression models of Predictors of Reported satisfaction of DHFF implementation by Facility in-charges.

	The institutional arrangements works well to control elements of corruption that may occur during procurement processes using funds received and managed at health facilities	The health facility have legal autonomy to receive and spend fund/resources received from different sources	DHFF facilitates functions of the distinct management	DHFF facilitate health facility spending smooth as a government institute	DHFF lead to improvement of management of health facility as independent entity
**Variable**	**AOR(CI)**	**AOR(CI)**	**AOR(CI)**	**AOR(CI)**	**AOR(CI)**
Gender					
Male	–	–	–	–	–
Female	0.99 (0.46-2.11)	0.84 (0.33-2.16)	0.62 (0.24-1.57)	0.62 (0.28-1.36)	0.78 (0.42-1.44)
Age	1.08 (1.03-1.13)***	1.06 (0.99-1.12)*	1.04 (1.01-1.08)**	1.07 (1.01-1.15)**	1.07 (1.03-1.12)**
Education					
Certificate	–	–	–	–	–
Diploma	2.15 (1.18-3.91)**	1.62 (0.83-3.13)	0.92 (0.48-1.76)	1.38 (0.74-2.59)	1.07 (0.51-2.25)
Advance diploma	4.33 (1.65-11.33)**	0.49 (0.11-2.06)	0.94 (0.29-2.98)	15 (0.51-467-12)	6.93 (0.34-138.08)
Degree	4.42 (0.65-29.76)	1.08 (0.36-3.16)	2.43 (0.40-14.65)	23.01(2.56-206.32)**	13.47(2.90-62.64)**
Cadre					
Laboratory technicians	–	–	–	–	–
Nurses	0.46 (0.12-1.77)	0.63 (0.25-1.56)	0.65 (0.22-1.91)	0.17 (0.03-0.83)**	0.14 (0.01-1.76)
Clinical officer	1.04 (0.35-3.09)	1.01 (0.32-3.17)	1.58 (0.45-5.48)	0.28 (0.04-1.79)	0.28 (0.01-4.49)
Medical officer	0.39 (0.05-2.92)	1.05 (0.21-5.31)	0.93 (0.13-6.29)	0.08 (0.01-0.42)**	0.10 (0.02-0.43)**
Experience (years)	0.91 (0.87-0.96)**	0.96 (0.90-1.03)	0.96 (0.91-1.02)	0.93 (0.85-1.01)*	0.95 (0.88-1.03)
Training sessions	1.04 (0.65-1.64)	0.81 (0.57-1.13)	1.15 (0.63-2.12)	1.42 (0.90-2.24)	1.70 (1.21-2.41)**
Facility level					
Dispensary	–	–	–	–	–
Health centre	0.37 (0.56-3.06)*	0.75 (0.35-1.59)	0.64 (0.21-1.95)	0.23 (0.09-0.57)**	0.28 (0.07-1.05)
Hospital	5.06 (0.58-43.70)	0.83 (0.02-26.05)	0.37 (0.02-4.94)	0.13 (0.01-3.60)	0.09 (0.01-0.72)
Locality					
Rural	–	–	–	–	–
Urban	1.31 (0.56-3.06)	2.26 (1.05-4.85)**	1.71 (0.72-4.06)	1.13 (0.48-2.66)	1.39 (0.49-3.92)
Number bed	1.03 (1.01-1.06)**	1.04 (1.01-1.07)**	1.03 (1.01-1.06)**	1.03 (1.01-1.06)**	1.03 (1.01-1.06)**
Number of staffs	0.97 (0.94-1.01)	0.97 (0.93-1.01)	0.96 (0.92-1.01)	0.96 (0.92-1.01)	0.97 (0.95-1.01)
Staffs trained on DHFF	1.26 (0.92-1.74)	1.35 (0.98-1.84)	1.30 (0.91-1.86)	1.39 (0.98-1.95)*	1.11 (0.84-1.44)

* p < 0.05** p < 0.01*** p < 0.001.

**Table 9:** Multivariate Ordered Logistic Regression models of Predictors of Reported Satisfaction of DHFF implementation by CHMT.

	The formula used to decide the amount health facility receives directly from ministry of finance works optimally	I am satisfied with the way DHFF is designed	DHFF has improved the facilities autonomy on managing all of its funds.	DHFF has facilitated health facilities receiving enough fund to cater all the planned activities	In general, DHFF is not performing as it was intended. (Reversed)
**Variable**	**AOR(CI)**	**AOR(CI)**	**AOR(CI)**	**AOR(CI)**	**AOR(CI)**
Gender					
Male	–	–	–	–	–
Female	1.27 (0.82-1.96)	0.87 (0.35-2.15)	0.77 (0.32-1.85)	0.79 (0.37-1.65)	0.76 (0.44-1.31)
Age	0.97 (0.94-1.01)	1.03 (0.98-1.08)	1.05 (1.01-1.09)**	0.95 (0.91-0.99)**	1.02 (0.97-1.06)
Education					
Certificate	–	–	–	–	–
Diploma	0.39 (0.16-0.95)**	0.84 (0.38-1.84)	1.36 (0.79-2.31)	1.07 (0.31-3.75)	9.06 (2.02-40.63)**
Advance diploma	0.74 (0.18-3.02)	2.43 (0.25-22.79)	4.20 (1.05-16.77)**	1.27 (0.24-6.65)	19.58 (3.48-109.86)**
Degree	0.57 (0.25-1.31)	0.75 (0.31-1.86)	1.43 (0.66-3.14)	0.86 (0.27-2.72)	10.65 (2.48-45.76)**
Master Degree	0.82 (0.05-11.75)	0.63 (0.07-5.75)	1.22 (0.09-16.48)	0.75 (0.14-3.90)	22.81 (5.29-98.16)***
Cadre					
Non-Medical	–	–	–	–	–
Nurses	1.31 (0.58-2.92)	0.93 (0.47-1.84)	1.41 (0.69-2.87)	1.76 (0.82-3.79)	0.78 (0.33-1.86)
Physician	0.94 (0.36-2.44)	0.43 (015-1.20)	1.05 (0.47-2.34)	1.14 (0.62-2.10)	0.72 (0.29-1.76)
Experience (years)	1.01 (0.96-1.05)	1.02 (0.93-1.11)	1.01 (0.95-1.06)	1.04 (0.97-1.11)	0.98 (0.89-1.07)
Trained on DHFF					
No	–	–	–	–	–
Yes	1.72 (0.45-6.56)	2.02 (0.78-5.24)	2.55 (1.18-5.49)**	1.57 (0.48-5.08)	1.06 (0.41-2.76)
Training on DHFF					
No	–	–	–	–	–
Yes	1.75 (0.28-10.93)	1.51 (0.31-7.46)	1.35 (0.32-5.60)	1.17 (0.66-5.18)	1.32 (0.33-5.19)

* p < 0.05** p < 0.01*** p < 0.001.

Facility in-charges who were medical officers were 86% less likely to be satisfied with the formula used to determine the disbursement amount (OR=0.14, CI = 0.03-0.68). In addition, more experienced facility in-charges were 10% less likely to be satisfied with the disbursement formula (OR=0.90, CI = 0.84-0.96). In contrast, CHMT members with diploma-level education were less likely to be satisfied with the formula (OR=0.39, CI = 0.16-0.95).

Interestingly, facility in-charges with a university degree-level were 16 times more satisfied with the DHFF design process (OR=16.12, CI = 1.88-142.89). In addition, as the age of the facility in-charges increased, there was an 8% higher likelihood of satisfaction with payment flexibility and resource management (OR=1.08, CI = 1.01-1.15) (Tables 7–9).

With regard to financial transparency, facility in-charges showed different levels of satisfaction depending on their age and educational background. Older facility in-charges were 6% more likely to be satisfied (OR=1.06, CI = 1.01-1.12), while those with a university degree were significantly more satisfied, being 6.45 times more likely to be satisfied (OR=6.45, CI = 1.66-25.09). However, experience seemed to moderate this satisfaction, as more experienced individuals were 8% less likely to be satisfied (OR=0.92, CI = 0.87-0.98). Similarly, facility in-charges with a university degree were 4.53 times more likely to be satisfied with how the DHFF had improved financial accountability (OR=4.53, CI = 1.17-17.54). Conversely, medical officer facility in-charges were 5% less likely to be satisfied with the DHFF’s payment flexibility and resource management (OR=0.05, CI = 0.01-0.21) (Tables 7 and 8).

Furthermore, as the age of CHMT members increased, there was slight disagreement about whether the DHFF had provided health facilities with sufficient funds for planned activities (OR=0.95, CI = 0.91-0.99). However, in terms of whether the DHFF was working as intended, CHMT members with different educational backgrounds were generally supportive of its effectiveness, with views tending to be positive for those with a diploma (OR=9.06, CI = 2.02-40.63), advanced diploma (OR=19.58, CI = 3.48-109.86), a university degree (OR=10.65, CI = 2.48-45.76) and master’s degree (OR=22.81, CI = 5.29-98.16) (Table 9).

Facility in-charges in health centres were 70% less likely to be satisfied with the design of the DHFF than those in dispensaries (OR=0.30, CI = 0.12 - 0.75). Furthermore, as the size of health facilities, measured by the number of beds, increased, managers were 3% more likely to be satisfied with the design of the DHFF (OR=1.03, CI = 1.01 - 1.06), and as more staff at these facilities were trained on DHFF, satisfaction were 36% higher. Similarly, in-charge from larger health facilities were 3% more likely satisfied with payment flexibility and resource management under the DHFF (OR=1.03, CI = 1.01-1.05), while an increase in staff numbers led to a 4% decrease in satisfaction (OR=0.96, CI = 0.93-0.99). However, as the number of trained staff increased, facility in-charges were 40% more likely to be satisfied with payment flexibility and resource management (OR=1.40, CI = 1.14-1.71).

Facility in-charges at health centres were 73% less likely to be satisfied with the improvement in financial transparency brought about by the DHFF (OR=0.27, CI = 0.11-0.69). Conversely, in-charges from larger facilities, as indicated by bed numbers, were 2% more likely to be satisfied with improvements in financial transparency resulting from DHFF implementation (OR=1.02, CI = 1.01-1.05), and those from facility with more trained staff showed a 41% increase in satisfaction (OR=1.41, CI = 1.12-1.76). Similarly, health centre facility in-charges were 76% less likely to be satisfied with improved financial accountability as a result of DHFF implementation (OR=0.24, CI = 0.09-0.59), while those from larger facilities showed a 3% increase in satisfaction with financial accountability (OR=1.03, CI = 1.01-1.06) (Table 7).

There was a notable increase in the perception of improved institutional arrangements to control elements of corruption in procurement processes as funds are received and managed in health facilities. Notably, the age of respondents was strongly correlated with reported perceptions. As age increased, facility managers were more likely to report control of corruption (OR=1.08, CI = 1.03 - 1.13), improved legal autonomy of the facility (OR=1.06, CI = 0.99 - 1.12), facilitation of clear management functions (OR=1.04, CI = 1.01 - 1.08), smooth spending as a government institution (OR=1.07, CI = 1.01 - 1.15), and management of the facility as an independent entity (OR=1.07, CI = 1.03 - 1.12) (Table 8). In addition, older CHMT had 5% higher odds of being satisfied with the improvement in the autonomy of managing funds (OR=1.05, CI = 1.01-1.09).

Considering education levels, CHMTs with advanced diplomas (OR=4.20, CI = 1.05-16.77) and those trained in DHFF (OR=2.55, CI = 1.18-5.49) generally had a more favorable view of facility autonomy in managing funds (Table 9). Compared to the reference group of laboratory technicians, nurses and medical officers were less satisfied with DHFF’s facilitation of smooth expenditure as a government institution, with medical officers expressing the lowest satisfaction. In addition, medical officers were significantly less likely to agree that the DHFF had led to an improvement in the management of health facilities as independent entities compared to laboratory technicians.

Facility type was also a significant factor, with health centres being less satisfied than dispensaries with respect to institutional arrangements that control elements of corruption in procurement processes. However, larger health facilities were more likely to have positive perceptions of the DHFF’s facilitation of corruption control, legal autonomy, clear management functions, smooth expenditure and improved facility management as an independent entity. In addition, managers in urban facilities were more likely to be satisfied with the legal autonomy of facilities to receive and spend funds/resources (OR=2.26, CI = 1.05-4.85) (Table 8).

## Discussion

This study investigated the implementation of the DHFF in Tanzania from the perspective of health care managers. We focused on the perceptions of both health facility in-charges and CHMT members regarding supportive supervision, fund management, resource availability, implementation capacity and the design of the DHFF.

The results of this study showed that the linkages between health facility in-charges and CHMT members played an enabling role in facilitating the operationalization of the DHFF. Overall, the study findings underscored that health manager’s perceived supportive supervision as instrumental in facilitating the implementation of the DHFF. This eased the burden of managing funds, which granted them greater financial autonomy. Respondents also noted a positive impact on the mobilization and availability of resources in health facilities. While expressing satisfaction with the overall design of the DHFF and its financial transparency, health managers expressed some reservations about the disbursement formula.

Although only a minority of respondents reported having received training on the implementation of the DHFF, routine supportive supervision, including orientation sessions, contributed to their additional knowledge and adherence to DHFF guidelines. Supportive supervision seems to facilitate the day-to-day operation of the DHFF, which is consistent with findings from other studies indicating that supportive supervision improves the adoption of health interventions similar to the implementation of the DHFF [26,31,44–46]. Unlike training and guidelines alone, engagement with senior management, and open discussions within health facility governing committees and community forums have been shown to enhance implementation success, improve program planning, achieve targets, and foster adaptation to local contexts [47–49]. The implementation of the DHFF has been instrumental in reducing the administrative burden associated with fund management. The implementers reported having DHFF guidelines, operational manuals and facility plans, which are carefully followed in the management of facility funds. Evidence suggests that while health facility in-charges oversee the flow of funds, decisions regarding planning, budgeting and procurement processes are made collaboratively, which is instrumental to successful policy uptakes [22,25]. Similar to previous studies, our findings indicate active participation by health facility in-charges, accountants and health facility management committees in running facilities [25]. Other studies show that the facility management teams work closely with health facility governing committees to address community health challenges and develop facility plans tailored to community needs, mostly funded via DHFF [21,22,25,50].

Health facility in-charges emphasized the importance of institutional autonomy and noted improvements in resource control, while acknowledging the challenge of having insufficient resources to meet all facility needs. Conversely, members of CHMTs perceived the DHFF as creating opportunities for health facilities to mobilize additional resources. Previous studies have documented that the implementation of the DHFF led to improved facility cash flow and increased availability of resources for the procurement of health commodities and enable effective resource management [16,30]. This study highlights the importance of adhering to guidelines on the allocation of funds disbursed through DHFF for example funds for drug procurement. According to DHFF guidelines, health facilities are required to allocate 50% of their revenues to the procurement of drugs and medical supplies [16]. However, as documented in other studies, the findings indicate resource constraints remain a challenge in ensuring the availability of health commodities as operation of health facility comprise of many competing priorities [5,51].

The finding that the DHFF has increased the financial autonomy of facilities, as favored by the CHMT, aligns with the primary objective of the initiative and is supported by senior management [27]. Previous studies have indicated that having greater facility autonomy yields positive effects on the health system, including increased capacity to procure necessary health commodities [13,14,16].

Consistent with findings from other studies, the performance of healthcare managers is linked to the capacity building they receive during program implementation [52,53]. This study demonstrates that trained healthcare managers exhibit a strong capacity to effectively manage DHFF. These results align with recommendations from other studies, which advocate for the development of individual and institutional capacities, skills, and competencies to facilitate the achievement of intended outcomes [54,55]. Nurses and clinical officers expressed positive opinions on DHFF governance. This is particularly significant as, in line with the current design and status, PHC facilities are led by clinical officers and nurses, serving as health facility in-charges within dispensaries [39,56]. Health facility in-charges overseeing facilities situated in urban areas generally perceived DHFF as performing well, although they highlighted challenges related to resource constraints, likely stemming from the high demand for care due to large population catchment areas. Several studies have indicated that patients tend to seek services from well-performing facilities, often located in urban settings where healthcare utilization rates are proportionally high, leading to observed resource limitations [57–60]. Continuous capacity building for frontline health managers is essential to ensure that the autonomy granted by DHFF can reduce dependency on the decisions and opinions of higher-level governance actors, thereby positively influencing local implementation practices [61].

This study revealed that nurses, clinical officers, and medical officers across various healthcare manager cadres hold a positive perception of DHFF’s contribution to healthcare efficiency. A similar finding was observed, indicating that the implementation of DHFF, aligned with the Facility Financial Accounting and Reporting System (FFARS), has improved financial management at the facility level [62]. Efficiency has been enhanced as resources from the health basket fund and complementary funds, such as insurances and user fees, are visibly tracked, leading to their optimal utilization in accordance with facility plans [16]. Slight differences were noted in the perceived availability of health services across different facility levels. In-charges at dispensaries recognized DHFF for increasing the availability of services, while those at health centres and hospitals perceived a decrease in service availability. The availability of services according to needs correlates with the resources accrued. Another study on DHFF documented a 40% decline in revenue for health centres compared to dispensaries [62] highlighting the need for further research in this area.

The DHFF disbursement formula was perceived to pose challenges, with managers at the dispensary level generally expressing more satisfaction with its design, payment flexibility, and resource management. Consistent with findings from other studies, this research highlights varying opinions on financial transparency based on respondents’ characteristics [22,26].

Managers with higher levels of education tend to perceive improved financial accountability. The CHMTs members noted that DHFF functions as intended. This study suggests that health managers’ control over resources at the facility level enhances planning and management, contributing to the liquidity of most primary healthcare facilities. Weak institutional leadership, however, results in poor jurisdictional outcomes, leading to lower development of disease prevention activities and limited reimbursement of services for underperforming populations, most of whom are serviced at the community level [3]. Perceptions of improved institutional arrangements to control elements of corruption in procurement processes, if linked to community involvement in facility plan approval as per PHC guidelines, could enhance resource availability and community access to healthcare. A recent study conducted in Tanzania highlighted the weak accountability of resources generated at health facilities, appearing to hinder the achievement of universal health coverage objectives [28]. Involving and holding local actors accountable may increase the legitimacy and fairness of priority-setting decisions [8,63].

The disparity between CHMTs favoring DHFF for resource improvement, as opposed to health facility in-charges, underscores the importance of establishing universal performance indicators for DHFF. Moreover, our current study reveals that a well-designed multi-level supervision program significantly influences the performance of implementing actors representing the community. Existing evidence suggests that decisions within the DHFF context are primarily made by facility in-charges and facility governing committees [21,22].

Clearly designed schemes, coupled with transparency and accountability among frontline healthcare managers, strengthen primary healthcare institutions by aligning them with communities’ health needs and the resources accrued. The findings from our study contribute to expanding knowledge in this area and emphasize the need for program designers to integrate community engagement into the current resource management framework at the facility level. Facilitating such engagement involves ensuring that the capacity of health facilities for service provision and availability matches communities’ needs, thereby simplifying supplementary resource mobilization efforts.

This study has several limitations worth noting. Firstly, there’s uncertainty regarding whether the sample analyzed truly represents the broader population of health managers in districts of the Kilimanjaro and Morogoro regions of Tanzania. Although all districts were purposively selected from these regions, participation in the survey was voluntary. Nonetheless, the study managed to achieve a substantial sample size, facilitated by mobile technology that allowed health managers to respond conveniently. This approach also resulted in a broad sampling across CHMTs members and health facility in-charges. Secondly, the study relies on self-reported measures, which may introduce biases and affect the accuracy of responses. This concern is particularly relevant in assessing experience with DHFF implementation guidelines, potentially skewing results. Thirdly, the absence of DHFF performance indicators in Tanzania limited our ability to validate findings regarding the perceived impact and satisfaction of CHMTs members and facility in-charges.

Despite these limitations, the study offers valuable insights into the health managers’ perspectives regarding the DHFF implementation in Tanzania.

## Conclusion

This study highlights the critical role of supportive supervision, financial autonomy and collaborative resource management in the successful implementation of the DHFF initiative in Tanzania. The findings show that effective linkages between health facility managers and CHMTs are critical to the operationalisation of DHFF, improving both resource mobilisation and utilisation at the facility level. While the overall design of the DHFF and its financial transparency have been well received, challenges remain, particularly around the disbursement formula and resource constraints, which vary across facility levels. The importance of continuous capacity building for health managers is emphasised, as it strengthens institutional autonomy and promotes efficient resource management in line with community health needs. The study highlights the need to integrate community engagement into the resource management framework to improve accountability and better align health service delivery with the needs of the population. These findings contribute to a broader understanding of the impact of the DHFF on primary health care, with implications for refining health financing models in similar contexts.

## Supporting information

S1 Checklist PLOS questionnaire on inclusivity in global research.

S1 Appendix **Table A:** Estimated Healthcare Manager Respondents. **Table B:** Frequencies and percentages of reported services provision (Facility). **Table C:** Health facility in-charge questionnaire. **Table D:** The Brant test for the proportional odds assumptions.

## Abbreviation

DHHF: Direct health facility financing
CHMT: Council health management team
DMOs: District medical officers
DHS: District health secretaries
PO-RALG: President office, ministry of regional and local government administration
SNF: Swiss National Science Foundation
ODK: the Open Data Kit
