# Supplementary material for: Healthcare managers’ perspectives on direct health facility financing in Tanzania
Source: PLOS Glob Public Health. 2025 May 28;5(5):e0003772. doi: 10.1371/journal.pgph.0003772 (PMC12118865; doi:10.1371/journal.pgph.0003772)
Supplement: S1 Appendix — Table A: Estimated Healthcare Manager Respondents. Table B: Frequencies and percentages of reported services provision (Facility). Table C: Health facility in-charge questionnaire. Table D: The Brant test for the proportional odds assumptions. [file pgph.0003772.s002.docx]

**Table A: Estimated Healthcare Manager Respondents**

|  | **Facility in-charge** | **CHMT** | **Total** |
| --- | --- | --- | --- |
| Total Managers | 561 | 288 | 849 |
| 50% Response | 280 | 144 | 424 |
| 25% Response | 140 | 72 | 212 |
| 5% response | 70 | 36 | 106 |

*Note: Kilimanjaro and Morogoro has 16 Districts in total*

**Table B: Frequencies and percentages of reported services provision (Facility)**

|  | **N (%)** | **Dispensary n=155** | **Health centre n=46** | **Hospital n=3** |
| --- | --- | --- | --- | --- |
| Facility provide services to NCDs patients | 185 (90.7) | 138 (89.0) | 44 (95.7) | 3 (100.0) |
| Special arrangement to provide NCDs services (OPD, NCD clinic day, NCDs wing) | 143 (77.3) | 100 (64.5) | 40 (86.9) | 3 (100.0) |
| Number of staff in a facility in total (mean)(Standard deviation) | 9.8 (17.6) | 3.8 (3.2) | 25 (23.1) | 82 (51.0) |
| Number of inpatient/observation beds (mean)(Standard deviation) | 8.4 (13.5) | 3.4 (2.9) | 22.1 (16.1) | 56 (49.0) |
| Enough building space to provide daily services | 53 (25.9) | 44 (28.4) | 9 (19.6) | 3 (100.0) |

**Table C: Health facility in-charge questionnaire**

| **Provider Background Questions: Demographic Information** | | | |
| --- | --- | --- | --- |
| A01 | A01. What is your sex? | 0 | Male |
|  |  | 1 | Female |
| A02 | A02. How old are you? |  | |
| A03 | A03.What is your highest level of education? |  | |
| A04 | A04.What is your cadre? *(healthcare manager/facility in-charge)* | 1 | Enrolled Nurse |
|  |  | 2 | Registered Nurse |
|  |  | 3 | Nurse Officer |
|  |  | 4 | Assistant Clinical Officer |
|  |  | 5 | Clinical Officer |
|  |  | 6 | Assistant Medical Officer |
|  |  | 7 | Medical Officer/Dental Officer |
|  |  | 8 | Medical specialist (MMED) |
|  |  | 9 | Pharmacist |
|  |  | 10 | Laboratory technician |
|  |  | 11 | Other |
| A05 | Please specify your cadre |  |  |
| A06 | A06.How many years have you served in this position? |  |  |
| A07. | A07.What are your management position? | 1. Facility in-charge | |
|  |  | 1. CHMT | |
| **Facility information: At the second level, the focus will be on the entire component relating to following rules, regulation, and autonomy of the institution and the availability of the services."** | | | |
| A08 | A08.Does the health facility you manage serve mostly rural or urban patients | 0 | Rural |
|  |  | 1 | Urban |
| A09 | Region | 1 | Kilimanjaro |
|  |  | 2 | Morogoro |
| A10 | District |  |  |
| A11. | A11.Which best describes the health facility you manage | 1 | Dispensary |
|  |  | 2 | Health Centre |
|  |  | 3 | Hospital |
| A12 | A12.Does your facility provide services to NCDs patients? | 0 | No |
|  |  | 1 | Yes |
| A13. | A13.Since your facility is designated to offer chronic non communicable services, does it have special arrangement in provision of that service? | 0 | No |
|  |  | 1 | Yes |
| A14. | A14.What are the special arrangements? | 1 | NCDs offered at OPD with other conditions |
|  |  | 2 | NCDs clinics day (Diabetes and HT) |
|  |  | 3 | NCDs offered every day on dedicated room |
| A15. | A15. What are the sources of resources you are using to cover NCDs services provision at your facility? | 1 | Revenue from out of pocket |
|  |  | 2 | Revenue from iCHF |
|  |  | 3 | Revenue from NHIF |
|  |  | 4 | Grants from government |
|  |  | 5 | Medical supplies granted through Medical store departments (MSD) |
| A16. | A16. In the last six month, have you used fund collected through DHFF to pay for medicine and supplies requirement from MSD? | 0 | No |
|  |  | 1 | Yes |
| A17. | A17. In the last six month, have you used fund collected through DHFF to pay for medicine and supplies requirement from Prime vendor? | 0 | No |
|  |  | 1 | Yes |
| A18. | A18.How many inpatient beds/observation beds does your facility have |  |  |
| A19. | A19. How many staff does your facility have in total |  |  |
| A20. | A20. Does the facility where you work provide the following services? | 1 | Diabetes |
|  |  | 2 | Hypertension |
|  |  | 3 | Cancer |
|  |  | 4 | respiratory diseases |
|  |  | 5 | kidney disease |
|  |  | 6 | Not providing NCDs services |
| **DHFF Background Questions: We would like to ask several questions about DHFF at the health facility where you are PRIMARILY engaged** | | | |
| B01 | B01.How many training/mentorship sessions have you received ***about*** DHFF implementation and governance in last 12 months? *(If not attended fill 0)* |  |  |
| B02 | B02. What were the topics during training/mentoring? | 1 | Understanding the flow of funds |
|  |  | 2 | Managing funds |
|  |  | 3 | Reporting writing |
|  |  | 4 | I do not know |
| B03 | B03. How many staff in your health facility have received training on implementation of DHFF |  |  |
| B04 | B04. What are the sources of funds that flow direct to the health facility account (DHFF) you are managing? | 1 | Ministry of finance (Health Basket fund) |
|  |  | 2 | District council own sources |
|  |  | 3 | NHIF Reimbursements |
|  |  | 4 | iCHF reimbursements |
|  |  | 5 | Cost Sharing |
|  |  | 6 | Receipt in Kind |
|  |  | 7 | Local Government capital Development Grant |
|  |  | 8 | Implementing partner’s money |
|  |  | 9 | Other (mention)----- |
| B05 | B05. Are the funds, earmarked for specific types of activities or interventions | 0 | No |
|  |  | 1 | Yes |
| B06 | B06. If yes, can you list the types of activities or interventions? |  |  |
| B07 | B07. Do you have Guidelines and operational manuals for DHFF and FFARS | 0 | No |
|  |  | 1 | Yes |
| B08 | B08. Have you ever convene a management meeting? | 0 | No |
|  |  | 1 | Yes |
| B09 | B09. How many times do you convene HF Management meetings and then give various response options per quarter? |  |  |
| B10 | B10. Do you have Annual Health Facility Plan Guideline? | 0 | No |
|  |  | 1 | Yes |
| B11 | B11. Do you have annual health facility plan - HFP? | 0 | No |
|  |  | 1 | Yes |
| B12 | B12. In case of any challenges in the course of implementing the program, where do you report first? | 1 | DMO |
|  |  | 2 | District DHFF coordinator |
|  |  | 3 | Others……(mention) |
| B13 | B13. When did you receive your last supportive supervision from CHMT? |  |  |
| C  B14 | B14. During the supportive supervision did you discuss issues of DHFF implementation? | 0 | No |
|  |  | 1 | Yes |
| B15 | B15. Do you involved other health care workers in DHFF program implementation decision making? | 0 | No |
|  |  | 1 | Yes |
| \| B16 \| \| --- \| \|  \| | B16. Did you receive the whole amount of funds as requested in your last Financial Year Budget? | 0 | No |
|  |  | 1 | Yes |
| B17 | B17. How frequently do you submit your Financial and Technical report on DHFF program implementation to the district level annually? |  |  |
| B18 | B18. Do you receive timely the funds from NHIF, iCHF, Basket fund, district grant? | 0 | No |
|  |  | 1 | Yes |
| B19 | B19. Does the disbursed of the funds to the health facility meet operational requirements as stipulated in the budget? | 0 | No |
|  |  | 1 | Yes |
| B20 | B20. Does the facility use government procurement systems to purchase supplies needed to service the health beneficiaries? | 0 | No |
|  |  | 1 | Yes |
| B21 | B21. Are the DHFF finance and accounting staff trained/oriented in accounting procedures, including the disbursement guidelines and reporting? | 0 | No |
|  |  | 1 | Yes |
| B22 | B22. Are the DHFF finance and accounting managing staff adequately qualified and experienced? | 0 | No |
|  |  | 1 | Yes |
| **Please answer the following to the best of your knowledge regarding the health facility you manage:** | | | |
| C01 | C01.Does the flow of funds direct to your facility decrease the funds administrative burden? | 0 | No |
|  |  | 1 | Yes |
| C02 | C02.Has the facility ever experienced problems in the past involving the receipt, accounting and/or administration of funds? | 0 | No |
|  |  | 1 | Yes |
| C03 | C03. Does the facilities has full autonomy on planning all activities you want implement? | 0 | No |
|  |  | 1 | Yes |
| C04 | C04. Does the facilities has full autonomy on budgeting? | 0 | No |
|  |  | 1 | Yes |
| C05 | C05. Does the facilities has full autonomy on managing all of its funds? | 0 | No |
|  |  | 1 | Yes |
| C06 | C06. Does the facility management have direct control of finances? | 0 | No |
|  |  | 1 | Yes |
| C07 | C07.Do you receive fund to cater all the planned activities? | 0 | No |
|  |  | 1 | Yes |
| C08 | C08. Do you purchase all services required on time? | 0 | No |
|  |  | 1 | Yes |
| C09 | C09. Do you get delays in deciding to purchase required input for services? | 0 | No |
|  |  | 1 | Yes |
| C10 | C10. How many weeks on average you delay to purchase the required inputs for services provision? |  |  |
| C11 | C11.Do you have all the medication required to provide services in every quarter? | 0 | No |
|  |  | 1 | Yes |
| C12 | C12.Do you have all the medical equipment required for provision of services in this facility? | 0 | No |
|  |  | 1 | Yes |
| C13 | C13.Do you have enough building space to provide daily services? | 0 | No |
|  |  | 1 | Yes |
| C14 | C14.What is the frequency of ordering medicine per quarter? |  |  |
| C15 | C15.For the last three times you ordered medicine, did you have enough fund to pay for the needs? | 0 | No |
|  |  | 1 | Yes |

| **We now present a series of statements and ask you to express your agreement on a scale from Strongly agree, to strongly disagree. This is your own personal opinion, and will be kept strictly anonymized so please be completely honest.** | | | | | | |
| --- | --- | --- | --- | --- | --- | --- |
| D01 | D01.The introduction of DHFF and its implementation to the moment in my place of work has improved efficiency in provision of services | strongly disagree | disagree | neutral | agree | strongly agree |
| D02 | D02.The introduction and implementation of DHFF creates an environment in which health facilities are more likely to respond to financial incentives | strongly disagree | disagree | neutral | agree | strongly agree |
| D03 | D03.DHFF has improved the availability of health services | strongly disagree | disagree | neutral | agree | strongly agree |
| D04 | D04.DHFF has improved mobilization of resources | strongly disagree | disagree | neutral | agree | strongly agree |
| D05 | D05. DHFF has facilitated financing of health services at health facilities | strongly disagree | disagree | neutral | agree | strongly agree |
| D06 | D06. The formular used to decide the amount health facility receives directly from ministry of finance works optimally | strongly disagree | disagree | neutral | agree | strongly agree |
| D07 | D07. I am satisfied with the way DHFF is designed | strongly disagree | disagree | neutral | agree | strongly agree |
| D08 | D08. DHFF system provides a flexible approach for health facilities payment and management of the resources | strongly disagree | disagree | neutral | agree | strongly agree |
| D09 | D09. DHFF has improved financial transparency | strongly disagree | disagree | neutral | agree | strongly agree |
| D10 | D10. DHFF has improved financial accountability | strongly disagree | disagree | neutral | agree | strongly agree |
| D11 | D11.The institutional arrangements works well to control elements of corruption that may occur during procurement processes using funds received and managed at health facilities | strongly disagree | disagree | neutral | agree | strongly agree |
| D12 | D12.The health facility have legal autonomy to receive and spend fund/resources received from different sources | strongly disagree | disagree | neutral | agree | strongly agree |
| D13 | D13. DHFF facilitates functions of the distinct management | strongly disagree | disagree | neutral | agree | strongly agree |
| D14 | D14. DHFF facilitate health facility spending smooth as a government institute | strongly disagree | disagree | neutral | agree | strongly agree |
| D15 | D15. DHFF lead to improvement of management of health facility as independent entity | strongly disagree | disagree | neutral | agree | strongly agree |
| D16 | D16. DHFF has improved the receiving of the fund | strongly disagree | disagree | neutral | agree | strongly agree |
| D17 | D17. Health facility faces trouble with the administrative issues that hamper the transfers of funds | strongly disagree | disagree | neutral | agree | strongly agree |
| D18 | D18. The organizational structure and governance is appropriate for the needs of the DHFF | strongly disagree | disagree | neutral | agree | strongly agree |

**Table D: The Brant test for the proportional odds assumptions**

| **Variable** | **Chi-square** | **p-value** | **Degrees of Freedom** |
| --- | --- | --- | --- |
| All | 78.12 | 0.16 | 27 |
| Gender | 8.59 | 0.13 | 3 |
| Age category | 0.93 | 0.81 | 3 |
| Education level | 21.16 | 0.26 | 3 |
| Marital status | 4.63 | 0.20 | 3 |
| Locality | 8.11 | 0.09 | 3 |
| Occupation | 4.01 | 0.26 | 3 |
| Health condition reported | 1.43 | 0.69 | 3 |
| Social protection | 6.55 | 0.08 | 3 |
| Facility level | 0.39 | 0.94 | 3 |
